# *catena*-Poly[[[di-μ_2_-hydroxido-bis­[(di-2-pyridylamine)nickel(II)]]-μ-fumarato] dihydrate]

**DOI:** 10.1107/S1600536809013580

**Published:** 2009-04-18

**Authors:** Jian Yu

**Affiliations:** a Department of Chemistry, Lishui University, 323000 Lishui, ZheJiang, People’s Republic of China.

## Abstract

The Ni^II^ ion in the one-dimensional title complex, {[Ni_2_(C_4_H_2_O_4_)(OH)_2_(C_10_H_9_N_3_)_2_]·2H_2_O}_*n*_, has a distorted square-pyramidal coordination environment formed by three O atoms from two bridging hydroxide groups and one carboxyl­ate group of the fumarate ligand and two pyridine N atoms from a di-2-pyridylamine (dpa) ligand. Two hydroxide groups link adjacent metal centers, forming a centrosymmetric four-membered [Ni_2_(OH)_2_] ring. In the crystal structure, the H atoms of the bridging hydroxide groups form inter­molecular hydrogen bonds to both water mol­ecules. These are further linked to the uncoordinated O atoms of the carboxyl­ate groups and the NH group of a dpa ligand to generate a three-dimensional network from the chains of the coordination polymer.

## Related literature

For applications of transition metal complexes with polypyridylamine ligands, see: Cotton *et al.* (1998 ▶). For details of complexes of bis­pyridine ligands, see: Liu *et al.* (2008 ▶). For the role of carboxyl­ate substituents in building coordination networks, see: Nathan & Traina (2003 ▶).
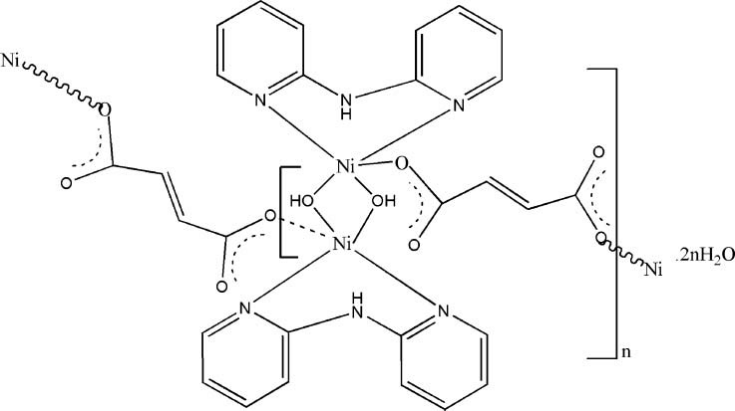


## Experimental

### 

#### Crystal data

[Ni_2_(C_4_H_2_O_4_)(OH)_2_(C_10_H_9_N_3_)_2_]·2H_2_O
*M* *_r_* = 643.92
Triclinic, 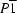

*a* = 8.135 (2) Å
*b* = 8.834 (3) Å
*c* = 10.015 (3) Å
α = 70.545 (4)°
β = 71.103 (4)°
γ = 75.785 (4)°
*V* = 634.3 (3) Å^3^
*Z* = 1
Mo *K*α radiation
μ = 1.55 mm^−1^
*T* = 298 K
0.22 × 0.18 × 0.12 mm

#### Data collection

Bruker APEXII area-detector diffractometer
Absorption correction: multi-scan (*SADABS*; Bruker, 2004 ▶) *T*_min_ = 0.727, *T*_max_ = 0.836
3238 measured reflections
2216 independent reflections
1984 reflections with *I* > 2σ(*I*)
*R*_int_ = 0.014

#### Refinement

*R*[*F* ^2^ > 2σ(*F* ^2^)] = 0.023
*wR*(*F* ^2^) = 0.063
*S* = 0.95
2216 reflections
190 parameters
4 restraints
H atoms treated by a mixture of independent and constrained refinement
Δρ_max_ = 0.30 e Å^−3^
Δρ_min_ = −0.35 e Å^−3^

### 

Data collection: *APEX2* (Bruker, 2004 ▶); cell refinement: *SAINT* (Bruker, 2004 ▶); data reduction: *SAINT*; program(s) used to solve structure: *SHELXS97* (Sheldrick, 2008 ▶); program(s) used to refine structure: *SHELXL97* (Sheldrick, 2008 ▶); molecular graphics: *ORTEPIII* (Burnett & Johnson, 1996 ▶) and *ORTEP-3 for Windows* (Farrugia, 1997 ▶); software used to prepare material for publication: *SHELXL97*.

## Supplementary Material

Crystal structure: contains datablocks I, global. DOI: 10.1107/S1600536809013580/sj2613sup1.cif

Structure factors: contains datablocks I. DOI: 10.1107/S1600536809013580/sj2613Isup2.hkl

Additional supplementary materials:  crystallographic information; 3D view; checkCIF report

## Figures and Tables

**Table 1:** Selected bond lengths (Å)

Ni1—O4	1.9671 (15)
Ni1—O4^i^	1.9713 (15)
Ni1—N1	1.9984 (18)
Ni1—N3	2.0314 (18)
Ni1—O1	2.2232 (16)
Ni1—Ni1^i^	2.9753 (8)

Symmetry code: (i) 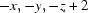.

**Table 2:** Hydrogen-bond geometry (Å, °)

*D*—H⋯*A*	*D*—H	H⋯*A*	*D*⋯*A*	*D*—H⋯*A*
O4—H4*A*⋯O2	0.83 (3)	2.068 (16)	2.848 (2)	158 (3)
O1*W*—H1*WA*⋯O2^ii^	0.840 (10)	1.912 (10)	2.752 (2)	178 (3)
O1*W*—H1*WB*⋯O4^iii^	0.85 (3)	1.92 (3)	2.765 (2)	172 (3)
N2—H2*B*⋯O1*W*^iv^	0.86	1.99	2.784 (3)	153

Symmetry codes: (ii) 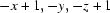; (iii) 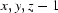; (iv) 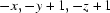.

**Fig. 1.**
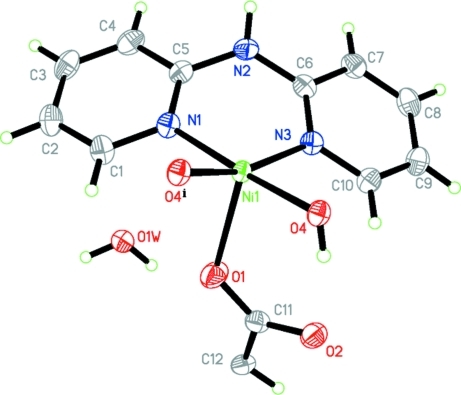
Part of the structure of the title compound showing the atom-labeling scheme [symmetry codes: (i) -x, -y, -z + 2]. Displacement ellipsoids are shown at the 30% probability level.

**Fig. 2.**
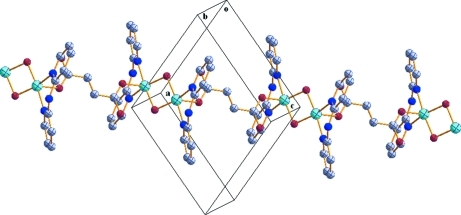
Partial packing diagram showing the formation of the one-dimensional zigzag chain.

## supplementary crystallographic information

### Comment

Transition metal complexes with polypyridylamine ligands have diverse structures and special optical and electromagnetic properties (Cotton *et al.*, 1998) and have aroused great interest among researchers. Multidentate amine ligands usually exhibit donor as well as acceptor properties and can be used as popular chelating ligands (Nathan & Traina, 2003).  On the other hand, carboxylates are attractive as metal-binding units in coordination networks because the negative charge significantly enhances their ability to bind strongly to metal centers, a feature which undoubtedly contributes to the robust nature of the resulting materials (Liu *et al.*, 2008). In this paper, we report the synthesis and crystal structure of the title compound (I), Figure 1.

The Ni1 atom in the title complex has a distorted square pyramidal coordination environment formed by one bidentate dpa ligand, two hydroxyl groups and one carboxylate group. The two peripheral pyridine N atoms from the dpa ligand [Ni1—N1 = 1.9984 (18)Å and Ni1—N3 =2.0314 (18) Å] and the two hydroxyl groups form the basal plane [Ni1—O4 =1.9713 (15) Å], the remaining apical position is occupied by an O atom of a carboxylate group from the fum^2-^ ligand [Ni1—O1 =2.2232 (16) Å], Table 1.  In addition, the inversion related hydroxyl groups link Ni(II) ions into a centrosymmetric [Ni_2_(OH)_2_]  four-membered ring. Furthermore, the fum^2-^ ligands bridge to an adjacent Ni^II^  linking the four-membered rings into a zigzag chain (Figure 2).

In the crystal structure, the H atoms of both water molecules and hydroxyl groups are involved in intermolecular hydrogen bonds with the O atoms of uncoordinated carboxylate groups and –NH group from dpa ligand which link the one-dimensional chains to form a three-dimensional network (Table 2).

### Experimental

Dpa (0.20 mg,0.1 mmol), Ni(CH_3_COO)2 (0.28 mg, 0.12 mmol) and Na_2_fum (0.22 mg, 0.09 mmol), were added to a mixture (12 mL) of methanol and acetonitrile (V/V=1:5). The solution was heated and stirred for two hours and was then kept at room temperature yielding green, block-like crystals over two weeks.

### Refinement

All H atoms attached to C and N atoms were fixed geometrically and treated as riding with C—H = 0.93 Å (ring) or N—H– 0.86 Å  with *U*_iso_(H) = 1.2*U*_eq_. H atoms of water molecule and hydroxyl group were located in difference Fourier maps and included in the subsequent refinement using restraints (O—H= 0.83 (1) Å) with *U*_iso_(H) = 1.5*U*_eq_(O).

### Crystal data

**Table:**

[Ni_2_(C_4_H_2_O_4_)(OH)_2_(C_10_H_9_N_3_)_2_]·2H_2_O	*Z* = 1
*M**_r_* = 643.92	*F*(000) = 332
Triclinic, *P*1	*D*_x_ = 1.686 Mg m^−^^3^
Hall symbol: -P 1	Mo *K*α radiation, λ = 0.71073 Å
*a* = 8.135 (2) Å	Cell parameters from 2216 reflections
*b* = 8.834 (3) Å	θ = 2.2–25.1°
*c* = 10.015 (3) Å	µ = 1.55 mm^−^^1^
α = 70.545 (4)°	*T* = 298 K
β = 71.103 (4)°	Block, green
γ = 75.785 (4)°	0.22 × 0.18 × 0.12 mm
*V* = 634.3 (3) Å^3^	

### Data collection

**Table:**

Bruker APEXII area-detector diffractometer	2216 independent reflections
Radiation source: fine-focus sealed tube	1984 reflections with *I* > 2σ(*I*)
graphite	*R*_int_ = 0.014
φ and ω scans	θ_max_ = 25.1°, θ_min_ = 2.2°
Absorption correction: multi-scan (*SADABS*; Bruker, 2004)	*h* = −9→9
*T*_min_ = 0.727, *T*_max_ = 0.836	*k* = −10→9
3238 measured reflections	*l* = −11→11

### Refinement

**Table:**

Refinement on *F*^2^	Primary atom site location: structure-invariant direct methods
Least-squares matrix: full	Secondary atom site location: difference Fourier map
*R*[*F*^2^ > 2σ(*F*^2^)] = 0.023	Hydrogen site location: inferred from neighbouring sites
*wR*(*F*^2^) = 0.063	H atoms treated by a mixture of independent and constrained refinement
*S* = 0.95	*w* = 1/[σ^2^(*F*_o_^2^) + (0.0374*P*)^2^ + 0.2972*P*] where *P* = (*F*_o_^2^ + 2*F*_c_^2^)/3
2216 reflections	(Δ/σ)_max_ = 0.001
190 parameters	Δρ_max_ = 0.30 e Å^−^^3^
4 restraints	Δρ_min_ = −0.35 e Å^−^^3^

### Special details

**Table:**

Geometry. All e.s.d.'s (except the e.s.d. in the dihedral angle between two l.s. planes) are estimated using the full covariance matrix. The cell e.s.d.'s are taken into account individually in the estimation of e.s.d.'s in distances, angles and torsion angles; correlations between e.s.d.'s in cell parameters are only used when they are defined by crystal symmetry. An approximate (isotropic) treatment of cell e.s.d.'s is used for estimating e.s.d.'s involving l.s. planes.
Refinement. Refinement of *F*^2^ against ALL reflections. The weighted *R*-factor *wR* and goodness of fit *S* are based on *F*^2^, conventional *R*-factors *R* are based on *F*, with *F* set to zero for negative *F*^2^. The threshold expression of *F*^2^ > σ(*F*^2^) is used only for calculating *R*-factors(gt) *etc*. and is not relevant to the choice of reflections for refinement. *R*-factors based on *F*^2^ are statistically about twice as large as those based on *F*, and *R*- factors based on ALL data will be even larger.

### Fractional atomic coordinates and isotropic or equivalent isotropic displacement parameters (Å^2^)

**Table:**

	*x*	*y*	*z*	*U*_iso_*/*U*_eq_	
Ni1	0.04021 (3)	0.15037 (3)	0.87985 (3)	0.02129 (10)	
N1	−0.0829 (2)	0.3069 (2)	0.73316 (19)	0.0298 (4)	
N2	−0.0529 (2)	0.5376 (2)	0.7868 (2)	0.0345 (4)	
H2B	−0.1179	0.6218	0.8116	0.041*	
N3	0.1739 (2)	0.3286 (2)	0.85495 (19)	0.0287 (4)	
O1	0.2311 (2)	0.0443 (2)	0.70594 (17)	0.0452 (4)	
C12	0.5204 (3)	−0.0107 (3)	0.5612 (2)	0.0325 (5)	
H12	0.6387	−0.0250	0.5576	0.039*	
O1W	0.1972 (2)	0.1399 (2)	0.22330 (18)	0.0381 (4)	
O4	0.13677 (18)	−0.00305 (18)	1.03993 (15)	0.0272 (3)	
C1	−0.1404 (3)	0.2457 (3)	0.6520 (2)	0.0369 (5)	
H1	−0.1120	0.1347	0.6615	0.044*	
C2	−0.2378 (3)	0.3391 (3)	0.5568 (3)	0.0423 (6)	
H2	−0.2765	0.2925	0.5039	0.051*	
C3	−0.2782 (3)	0.5056 (3)	0.5407 (3)	0.0440 (6)	
H3	−0.3457	0.5719	0.4776	0.053*	
C4	−0.2178 (3)	0.5707 (3)	0.6181 (3)	0.0396 (6)	
H4	−0.2429	0.6819	0.6076	0.048*	
C5	−0.1178 (3)	0.4688 (3)	0.7134 (2)	0.0304 (5)	
C6	0.1043 (3)	0.4872 (3)	0.8252 (2)	0.0301 (5)	
C7	0.1878 (3)	0.6062 (3)	0.8296 (3)	0.0376 (5)	
H7	0.1352	0.7144	0.8122	0.045*	
C8	0.3477 (3)	0.5600 (3)	0.8600 (3)	0.0420 (6)	
H8	0.4049	0.6367	0.8643	0.050*	
C9	0.4246 (3)	0.3974 (3)	0.8844 (3)	0.0393 (6)	
H9	0.5349	0.3642	0.9026	0.047*	
C10	0.3345 (3)	0.2878 (3)	0.8812 (2)	0.0336 (5)	
H10	0.3861	0.1792	0.8978	0.040*	
C11	0.3878 (3)	−0.0135 (3)	0.7062 (2)	0.0324 (5)	
O2	0.4437 (2)	−0.0761 (2)	0.81955 (17)	0.0375 (4)	
H1WB	0.168 (4)	0.101 (4)	0.169 (3)	0.080*	
H1WA	0.3066 (15)	0.122 (4)	0.212 (4)	0.080*	
H4A	0.231 (3)	−0.046 (4)	0.995 (3)	0.080*	

### Atomic displacement parameters (Å^2^)

**Table:**

	*U*^11^	*U*^22^	*U*^33^	*U*^12^	*U*^13^	*U*^23^
Ni1	0.02217 (15)	0.02005 (15)	0.02074 (14)	−0.00466 (10)	−0.00701 (10)	−0.00218 (10)
N1	0.0313 (10)	0.0283 (10)	0.0282 (9)	−0.0074 (8)	−0.0095 (8)	−0.0020 (8)
N2	0.0349 (10)	0.0271 (10)	0.0419 (11)	0.0016 (8)	−0.0124 (9)	−0.0125 (8)
N3	0.0310 (10)	0.0258 (10)	0.0280 (9)	−0.0063 (8)	−0.0081 (7)	−0.0041 (8)
O1	0.0312 (9)	0.0669 (12)	0.0337 (9)	0.0070 (8)	−0.0072 (7)	−0.0209 (9)
C12	0.0292 (11)	0.0306 (12)	0.0344 (11)	0.0016 (9)	−0.0073 (9)	−0.0103 (10)
O1W	0.0398 (9)	0.0352 (9)	0.0442 (10)	−0.0011 (7)	−0.0164 (8)	−0.0153 (8)
O4	0.0272 (8)	0.0269 (8)	0.0266 (7)	−0.0062 (6)	−0.0091 (6)	−0.0031 (6)
C1	0.0461 (14)	0.0333 (13)	0.0332 (12)	−0.0124 (11)	−0.0153 (11)	−0.0025 (10)
C2	0.0474 (15)	0.0483 (15)	0.0354 (13)	−0.0157 (12)	−0.0177 (11)	−0.0045 (11)
C3	0.0381 (13)	0.0496 (16)	0.0384 (13)	−0.0048 (11)	−0.0190 (11)	0.0024 (12)
C4	0.0355 (13)	0.0334 (13)	0.0412 (13)	−0.0023 (10)	−0.0114 (11)	−0.0003 (11)
C5	0.0272 (11)	0.0302 (12)	0.0292 (11)	−0.0058 (9)	−0.0048 (9)	−0.0041 (9)
C6	0.0345 (12)	0.0298 (12)	0.0243 (10)	−0.0090 (9)	−0.0044 (9)	−0.0057 (9)
C7	0.0472 (14)	0.0295 (12)	0.0362 (12)	−0.0086 (11)	−0.0107 (11)	−0.0078 (10)
C8	0.0496 (15)	0.0415 (15)	0.0423 (13)	−0.0210 (12)	−0.0125 (11)	−0.0108 (11)
C9	0.0346 (13)	0.0443 (15)	0.0411 (13)	−0.0139 (11)	−0.0123 (11)	−0.0066 (11)
C10	0.0308 (12)	0.0336 (12)	0.0348 (12)	−0.0074 (10)	−0.0083 (9)	−0.0061 (10)
C11	0.0325 (12)	0.0312 (12)	0.0327 (12)	−0.0020 (10)	−0.0068 (10)	−0.0118 (10)
O2	0.0350 (9)	0.0420 (10)	0.0322 (8)	0.0022 (7)	−0.0102 (7)	−0.0108 (7)

### Geometric parameters (Å, °)

**Table:**

Ni1—O4	1.9671 (15)	O4—Ni1^i^	1.9713 (15)
Ni1—O4^i^	1.9713 (15)	O4—H4A	0.83 (3)
Ni1—N1	1.9984 (18)	C1—C2	1.364 (3)
Ni1—N3	2.0314 (18)	C1—H1	0.9300
Ni1—O1	2.2232 (16)	C2—C3	1.392 (4)
Ni1—Ni1^i^	2.9753 (8)	C2—H2	0.9300
N1—C5	1.347 (3)	C3—C4	1.362 (4)
N1—C1	1.355 (3)	C3—H3	0.9300
N2—C5	1.377 (3)	C4—C5	1.397 (3)
N2—C6	1.381 (3)	C4—H4	0.9300
N2—H2B	0.8600	C6—C7	1.404 (3)
N3—C6	1.347 (3)	C7—C8	1.365 (3)
N3—C10	1.354 (3)	C7—H7	0.9300
O1—C11	1.252 (3)	C8—C9	1.392 (4)
C12—C12^ii^	1.313 (4)	C8—H8	0.9300
C12—C11	1.499 (3)	C9—C10	1.364 (3)
C12—H12	0.9300	C9—H9	0.9300
O1W—H1WB	0.85 (3)	C10—H10	0.9300
O1W—H1WA	0.840 (10)	C11—O2	1.263 (3)
			
O4—Ni1—O4^i^	81.87 (6)	N1—C1—H1	118.4
O4—Ni1—N1	173.85 (7)	C2—C1—H1	118.4
O4^i^—Ni1—N1	94.01 (7)	C1—C2—C3	118.5 (2)
O4—Ni1—N3	94.06 (7)	C1—C2—H2	120.8
O4^i^—Ni1—N3	161.16 (7)	C3—C2—H2	120.8
N1—Ni1—N3	88.38 (7)	C4—C3—C2	119.5 (2)
O4—Ni1—O1	94.61 (6)	C4—C3—H3	120.3
O4^i^—Ni1—O1	101.31 (7)	C2—C3—H3	120.3
N1—Ni1—O1	90.68 (7)	C3—C4—C5	119.4 (2)
N3—Ni1—O1	97.33 (7)	C3—C4—H4	120.3
O4—Ni1—Ni1^i^	40.99 (4)	C5—C4—H4	120.3
O4^i^—Ni1—Ni1^i^	40.88 (4)	N1—C5—N2	119.92 (19)
N1—Ni1—Ni1^i^	134.72 (5)	N1—C5—C4	121.6 (2)
N3—Ni1—Ni1^i^	132.37 (5)	N2—C5—C4	118.5 (2)
O1—Ni1—Ni1^i^	100.55 (5)	N3—C6—N2	120.05 (19)
C5—N1—C1	117.87 (19)	N3—C6—C7	122.1 (2)
C5—N1—Ni1	124.23 (15)	N2—C6—C7	117.8 (2)
C1—N1—Ni1	117.85 (15)	C8—C7—C6	118.8 (2)
C5—N2—C6	127.58 (19)	C8—C7—H7	120.6
C5—N2—H2B	116.2	C6—C7—H7	120.6
C6—N2—H2B	116.2	C7—C8—C9	119.6 (2)
C6—N3—C10	117.40 (19)	C7—C8—H8	120.2
C6—N3—Ni1	122.94 (15)	C9—C8—H8	120.2
C10—N3—Ni1	119.42 (15)	C10—C9—C8	118.5 (2)
C11—O1—Ni1	123.67 (14)	C10—C9—H9	120.8
C12^ii^—C12—C11	124.0 (3)	C8—C9—H9	120.8
C12^ii^—C12—H12	118.0	N3—C10—C9	123.5 (2)
C11—C12—H12	118.0	N3—C10—H10	118.2
H1WB—O1W—H1WA	112 (2)	C9—C10—H10	118.2
Ni1—O4—Ni1^i^	98.13 (6)	O1—C11—O2	125.1 (2)
Ni1—O4—H4A	103 (2)	O1—C11—C12	118.0 (2)
Ni1^i^—O4—H4A	111 (3)	O2—C11—C12	116.88 (19)
N1—C1—C2	123.1 (2)		

Symmetry codes: (i) −*x*, −*y*, −*z*+2; (ii) −*x*+1, −*y*, −*z*+1.

### Hydrogen-bond geometry (Å, °)

**Table:**

*D*—H···*A*	*D*—H	H···*A*	*D*···*A*	*D*—H···*A*
O4—H4A···O2	0.83 (3)	2.07 (2)	2.848 (2)	158 (3)
O1W—H1WA···O2^ii^	0.84 (1)	1.91 (1)	2.752 (2)	178 (3)
O1W—H1WB···O4^iii^	0.85 (3)	1.92 (3)	2.765 (2)	172 (3)
N2—H2B···O1W^iv^	0.86	1.99	2.784 (3)	153

Symmetry codes: (ii) −*x*+1, −*y*, −*z*+1; (iii) *x*, *y*, *z*−1; (iv) −*x*, −*y*+1, −*z*+1.
